# Analysis of Single Nucleotide-Mutated Single-Cancer Cells Using the Combined Technologies of Single-Cell Microarray Chips and Peptide Nucleic Acid-DNA Probes

**DOI:** 10.3390/mi11070628

**Published:** 2020-06-27

**Authors:** Hajime Shigeto, Eriko Yamada, Mizuki Kitamatsu, Takashi Ohtsuki, Akira Iizuka, Yasuto Akiyama, Shohei Yamamura

**Affiliations:** 1 Health and Medical Research Institute, National Institute of Advanced Industrial Science and Technology (AIST), 2217-14 Hayashi-cho, Takamatsu, Kagawa 761-0395, Japan; e-yamada@aist.go.jp; 2 Department of Applied Chemistry, Faculty of Science and Engineering, Kindai University, 3-4-1 Kowakae, Higashi-Osaka, Osaka 577-8502, Japan; kitamatu@apch.kindai.ac.jp; 3 Department of Interdisciplinary Science and Engineering in Health Systems, Okayama University, 3-1-1 Tsushimanaka, Okayama 700-8530, Japan; ohtsuk@okayama-u.ac.jp; 4 Immunotherapy Division, Shizuoka Cancer Center Research Institute, Shizuoka 411-8777, Japan; a.iizuka@scchr.jp (A.I.); y.akiyama@scchr.jp (Y.A.)

**Keywords:** single-cell analysis, peptide nucleic acid (PNA) probe, cell microarray, single nucleotide mutation, T790M mutation, lung cancer, epidermal growth factor receptor (EGFR)

## Abstract

Research into cancer cells that harbor gene mutations relating to anticancer drug-resistance at the single-cell level has focused on the diagnosis of, or treatment for, cancer. Several methods have been reported for detecting gene-mutated cells within a large number of non-mutated cells; however, target single nucleotide-mutated cells within a large number of cell samples, such as cancer tissue, are still difficult to analyze. In this study, a new system is developed to detect and isolate single-cancer cells expressing the T790M-mutated epidermal growth factor receptor (EGFR) mRNA from multiple non-mutated cancer cells by combining single-cell microarray chips and peptide nucleic acid (PNA)-DNA probes. The single-cell microarray chip is made of polystyrene with 62,410 microchambers (31-40 µm diameter). The T790M-mutated lung cancer cell line, NCI-H1975, and non-mutated lung cancer cell line, A549, were successfully separated into single cells in each microchambers on the chip. Only NCI-H1975 cell was stained on the chip with a fluorescein isothiocyanate (FITC)-conjugated PNA probe for specifically detecting T790M mutation. Of the NCI-H1975 cells that spiked into A549 cells, 0–20% were quantitatively analyzed within 1 h, depending on the spike concentration. Therefore, our system could be useful in analyzing cancer tissue that contains a few anticancer drug-resistant cells.

## 1. Introduction

Single-cell analysis offers great potential for understanding the complex biology of various diseases and can also assist with diagnosis. Many single-cell-level analysis tools and systems are currently being developed [1,2,3]. In particular, microchip technology, especially the microchip system for processing cells, called cell chips, could potentially be a powerful tool for the easy, rapid, accurate, and highly sensitive analysis of target single cells that exist within a large number of different cells. Many cell chips with types of microarray [4,5,6,7,8,9] and microfluidic [10,11,12,13] have been reported for single-cells analysis. In particular, cell microarray chips are useful for high-throughput screening and analysis for cells. The fluorescent labeled antibodies [14,15,16,17,18] or fluorescent labeled DNA-based probes [19,20,21,22,23,24,25,26] are commonly used to screen for and analyze target cells. Although these probes have high sensitivity and specificity, it is difficult to detect slightly expressed proteins or the few nucleotide-mutated genes. In addition, it is more difficult to analyze these targets at single cells level. Recently, the screening and detection of anticancer drug-resistant cancer cells harboring single nucleotide-mutated genes has focused on cancer diagnosis [27,28,29]; therefore, we aimed to detect and isolate the single cells expressing the single nucleotide-mutated mRNA from multiple non-mutated cancer cells using our original cell chip technology and peptide nucleic acid (PNA)-based probes with high specificity.

In this study, lung cancer cells were used as a model to analyze the single nucleotide-mutated cancer cells. Lung cancer cells harbor various gene mutations in the epidermal growth factor receptor (EGFR) gene. Tyrosine kinase inhibitor (TKI), represented by Gefitinib, is a molecular-targeting anticancer drug that binds to the tyrosine kinase domain of the EGFR protein [30,31,32]. Gefitinib inhibits the signal transduction of the epidermal growth factor signal and induces cell death [33]. It is reported that cancer cells with the EGFR gene mutation (in particular, exon19del E746-A750 and L858R) respond to Gefitinib [31,32,33,34,35]. However, long-term administration of Gefitinib induces TKI-resistant cells. These cells often carry the T790M-mutation [36,37,38,39]. T790M-mutated EGFR protein loses its binding affinity with Gefitinib and becomes resistant to TKI [40]. Therefore, analysis of the composition ratio or the number of T790M-mutated cancer cells is necessary for the diagnosis and efficient treatment of lung cancer. A DNA-sequencing system is commonly used when analyzing EGFR gene mutation; in particular, the next-generation sequencer (NGS) excels at providing accurate analysis [41,42]. However, at least 20% of cancer cells in a cell sample must contain the target mutation [43,44,45]. Therefore, the DNA-sequencing system is not suitable for early diagnosis, at which point only a small number of mutated cancer cells are present. Although real-time PCR-based analyzing systems have high sensitivity, they also require that 5–10% or more of the total cancer cell samples harbor the target mutation [46,47]. These conventional methods also require expensive equipment, time-consuming detection (3–5 h for typical PCR systems), and expert technical knowhow. Image analysis is a promising method for detecting a small number of mutated cancer cells; however, it is difficult to analyze mutated cells at the single-cell level using general antibodies or other probes. In a previous study, we reported the novel fluoresce labeled PNA-DNA-based probes for the image analysis of three EGFR-mutated genes (exon19del E746-A750, L858R, and T790M) [48]. Using the PNA-DNA probes, we succeeded in specifically detecting EGFR-mutated cells. However, because of the limited number of mutated cancer cells analyzed using the normal slide-glasses or microwell-plates formats, it is difficult to calculate the ratio or detect an accurate number of rare mutated cancer cells contained within multiple cells.

In this study, we have developed a new detection system for single nucleotide-mutated cancer cells at the single-cell level using our original techniques combined with a single-cell microarray chip [7] and PNA-DNA probes [48]. After the T790M-mutated lung cancer cell line (NCI-H1975), as a model of anticancer drug-resistant cancer cells, was spiked into non-mutated cancer cells (A549), all cancer cells were separated into single cells using the single-cell microarray chip. Target T790M-mutated cancer cells (NCI-H1975) were discriminated from the non-mutated cancer cells (A549) using PNA-DNA probes on the chip.

## 2. Materials and Method

### 2.1. Construction of a Single-Cell Microarray Chip

**Figure 1:**
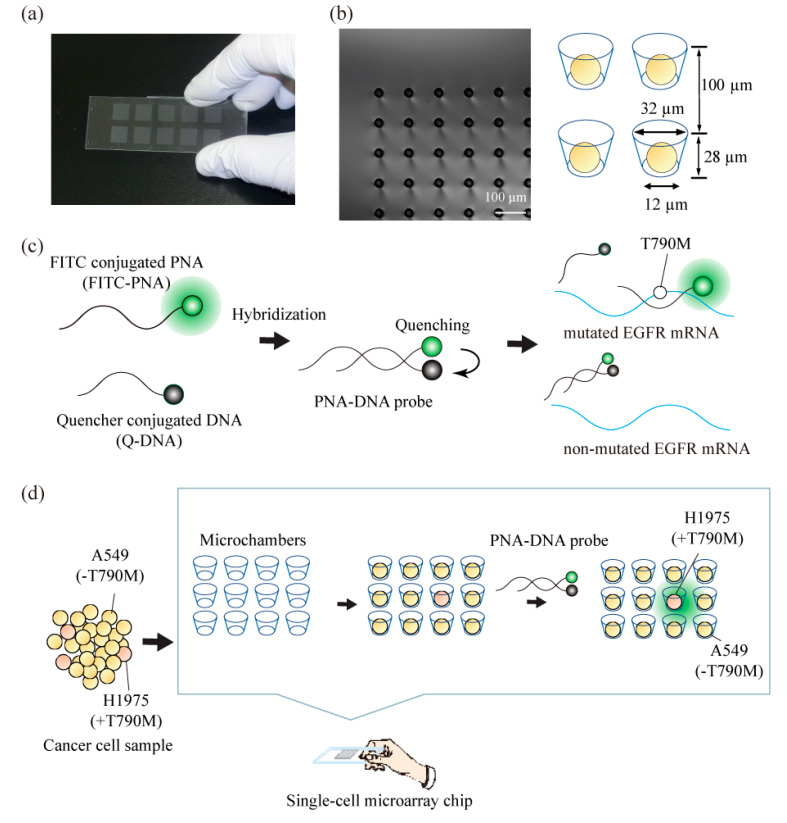
Schematic concept for the analysis of single nucleotide-mutated cancer cells. (**a**) Photograph of a real single-cell microarray chip device, which was made with polystyrene and consists of 62,410 microchambers (upper diameter: 31–40 µm, lower diameter: 11–20 µm, depth: 28 µm, pitch: 100 µm). Each single-cell microarray chip consisted of 10 different-sized patterns of clusters, and each cluster consisted of 6241 (79 × 79) microchambers. Each microchamber had the shape of a circular cone frustum. (**b**) Microscopic image of microchambers (upper diameter: 32 µm, lower diameter: 12 µm) of one cluster in the single-cell microarray chip. The picture shows single lung cancer cells occupying each microchamber. (**c**) Principle of target single nucleotide-mutated epidermal growth factor receptor (EGFR) mRNA detection with a peptide nucleic acid (PNA)-DNA probe. (**d**) Schematic process for the analysis of mutation-harboring cancer cells at the single-cell level using our original techniques combined with a single-cell microarray chip and a PNA-DNA probe.

The single-cell microarray chip consisted of 62,410 microchambers, with an upper diameter of 31–40 µm, a lower diameter of 11–20 µm, a depth of 28 µm, and a pitch of 100 µm. It was made of polystyrene using an ultraviolet-lithographie galvanoformung abformung (UV-LIGA) process designed by SEIKOH GIKEN Co. Ltd., Chiba, Japan (Figure 1a). Using UV-lithography, a photoresist substrate was exposed to and patterned with a metal photomask on a glass plate. The resulting patterned photoresist substrate was used to create a nickel mold with microstructures that were generated by electroforming. Finally, the polystyrene microarray chip was fabricated from the nickel mold using injection molding. Each microarray chip consisted of 10 different-sized patterns of clusters, and each cluster consisted of 6241 (79 × 79) microchambers. Each microchamber had the shape of a circular cone frustum and was able to accommodate cells.

### 2.2. Chip Surface Treatment

The surface of the single-cell microarray chip was hydrophilized with an oxygen plasma treatment using soft plasma-etching equipment (SEDE-PFA, Meiwafosis Co., Ltd., Tokyo, Japan), which made single-cell separation and analysis easier. The exposure time and electric current for plasma etching were described in previous studies [7].

### 2.3. Construction of PNA-DNA Probes

The detailed structure and principles of the target detection of PNA-DNA probes were described in previous reports [48]. Briefly, fluorescein isothiocyanate (FITC)-conjugated PNA (FITC-PNA; fluorescein-O-Linker-PNA(CTGCATGATG)-k-k; Panagene, Daejeon, Korea) and quencher-conjugated DNA (Q-DNA; TCATGCAG-Dabcyl; Integrated DNA Technologies, Coralville, IA, United States) were prepared. PNA-DNA probes were constructed by the hybridization of the synthesized FITC-PNA and Q-DNA. FITC-PNA and Q-DNA (10 µM final) were mixed in a PBS solution and self-assembled with an annealing program, as follows. The mixed reagent was heated at 95 °C for 5 min for denaturing. To hybridize the FITC-PNA and Q-DNA, the temperature was gradually decreased to 30 °C at the rate of −1.38 °C per minute. Finally, the mixture was incubated at 30 °C for 10 min and kept at 10 °C. The product was used directly in further experiments.

### 2.4. Cell Culture and Preparation

Human lung carcinoma cells lines, NCI-H1975 and A549 (ATCC, Manassas, VA, United States), were cultured in RPMI 1640 (Nakalai Tesque, Kyoto, Japan), containing 10% fetal bovine serum and antibiotics (100-U/mL penicillin–streptomycin (Gibco, Life Technologies Corporation, CA, United States)), and were subsequently harvested using trypsin. The various concentrations (0%, 5%, 10%, 20%) of NCI-H1975 and A549 suspension were each prepared at a concentration of 1.0–1.2 × 10^7^ cells/mL of medium. Each cell sample (500 µL) was used for analysis on the single-cell microarray chip.

### 2.5. Single-Cell Separation, Staining, and Analysis

To assess the single-cell confinement of cultured cells in the microchambers, we examined cell suspensions containing various concentrations of NCI-H1975 and A549. First, 500-µL RPMI medium of 1.0–1.2 × 10^7^ cells/mL was dispersed onto the single-cell microarray chip, followed by incubation for 30 min to allow cells to settle into the microchambers by gravity. Then, excess cells were removed from the chip surface by gentle washing with PBS. The microchambers were then examined with light images from an inverted fluorescence microscope (IX80, Olympus, Tokyo, Japan). To detect the EGFR-mutated single NCI-H1975 cells held in the microchambers, nuclei, cytokeratin (CK), and target T790M-mutated EGFR mRNA were stained with 1:1200 diluted 4,6-diamidino-2-phenylindole (DAPI) (Dojindo Laboratories Co., Kumamoto, Japan), 0.3-µM phycoerythrin (PE)-labeled Mouse Anti-Human CK clone CAM5.2 (BD Biosciences, San Jose, CA, United States), and a 1.5-µM final concentration of prepared PNA-DNA probe in 0.05% saponin/PBS, and allowed to react for 30 min, following which the chip surface was washed with PBS. Each single-cell microarray chip was visualized under the 20× objective lens of an Olympus FV3000 confocal laser microscope (Olympus, Tokyo, Japan). The FITC image was taken with a 488-nm laser with a variable barrier filter set at 500–600 nm. The DAPI image was obtained using a 405-nm laser with a variable barrier filter set at 430–470 nm. The CK image was obtained using a 562-nm laser with a variable barrier filter set at 600–670 nm.

## 3. Results and Discussion

### 3.1. Separation and Analysis of the Different Lung Cancer Cell Lines on the Single-Cell Microarray Chip

**Figure 2:**
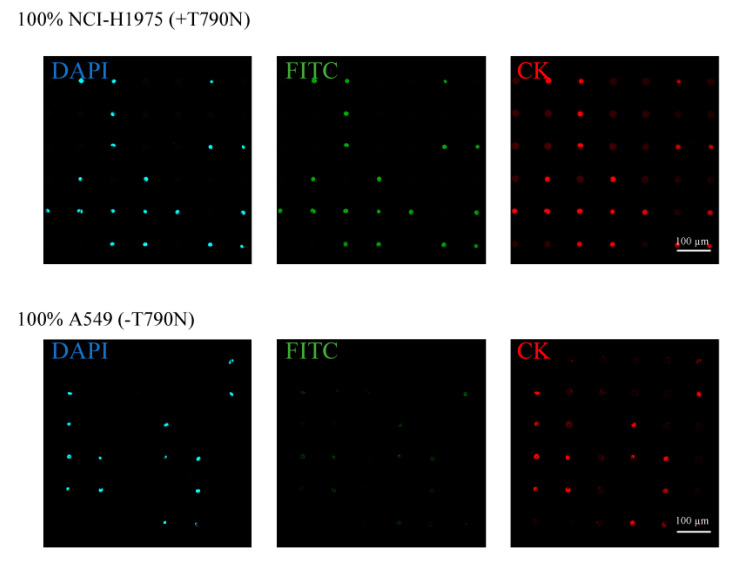
Separation and analysis of different lung cancer cell lines (NCI-H1975 and A549) on the single-cell microarray chip. The upper and lower figures show the representative fluorescent images of NCI-H1975 (T790M-mutated) and A549 (non-mutated) cells, respectively, stained by diluted 4,6-diamidino-2-phenylindole (DAPI; blue), fluorescein isothiocyanate (FITC)-conjugated peptide nucleic acid (PNA)-DNA probes (green), and phycoerythrin (PE)-labeled cytokeratin (CK) antibody (red).

To investigate the possibility of analyzing single nucleotide-mutated cancer cells at the single cell level, we analyzed the ratio or the exact number of gene-mutated cancer cells using the combined technologies of single-cell microarray chips and PNA-DNA probes. As a model of anticancer drug-resistant cancer cells, a lung cancer cell line, NCI-H1975, which harbors the T790M mutation, the resistance factor against anticancer drugs, was screened from multiple non-mutated lung cancer cell lines, A549. The single-cell microarray chip used in this study had 62,410 microchambers, which had an upper diameter of 31–40 µm, a lower diameter of 11–20 µm, a depth of 28 µm, and a pitch of 100 µm (Figure 1a,b). In our previous study [7], the NCI-H1650 lung cancer cell line was successfully separated into single cells using microchambers with an upper diameter of 32 µm and a lower diameter of 12 µm of one cluster in the chip. As the NCI-H1975 and A549 cell lines used in this study were approximately the same size as the NCI-H1650 cell line, microchambers with a 32-µm upper diameter and a 12-µm lower diameter were used for separating them into single cells. Dispersing the cancer cell suspension into the single-cell microarray chip resulted in NCI-H1975 and A549 occupying approximately 50% and 60% of microchambers on the chip, respectively (Figure 2). The two types of lung cancer cells were successfully separated into single cells and aligned on a single-cell microarray chip in the same way. These results indicate that, even if the two types of cancer cells (single nucleotide-mutated and non-mutated cells) are mixed, it is possible to separate them and determine the exact ratio of target mutation-harboring cancer cells on the single-cell microarray chip.

This study used DAPI for staining the cellular nucleus, PE-labeled anti-CK antibody for staining the CK, and an FITC-conjugated PNA-DNA probe for staining the T790M-mutated EGFR mRNA. PNA has high target binding ability due to PNA’s lack of electronic charge in the backbone and has the high specificity [49,50,51,52]. These PNA probes are expected to be more suitable than DNA probes for gene mutation analysis of single-nucleotide mutations. In a previous study [48], we developed the PNA-DNA probes for as an easy and rapid detection for EGFR gene mutations, such as exon19del E746-A750, T790M, and L858R. The developed PNA-DNA probes showed increases in fluorescence intensity against dose-dependent, in vitro target EGFR-mutated sequences, which indicates that FITC-PNA probes are separated from Q-DNA probes, and specifically bind to the target sequence of mutated EGFR mRNA (Figure 1b). To analyze the T790M-mutated cancer cell on the single-cell microarray chip, staining dyes were introduced into the cancer cells using the surfactant saponin after the cell suspension was dispersed onto the cell chip. The fluorescence of the stained cancer cells was observed under a confocal-laser scanning microscope. The DAPI and CK antibodies displayed a similar fluorescence intensity in every cancer cell. As antibody or nuclear staining could be successfully performed on the single-cell microarray chip, it was demonstrated that multiple staining was also possible on the microarray chip. The mean FITC fluorescence intensity in single cells was calculated using imaging software HCImage (Hamamatsu Corporation, Bridgewater, NJ, United States). The fluorescence intensities of NCI-H1975 (T790M-mutated cell) and A549 (non-mutated cell) were 116.6 and 53.5, respectively (Figure 2). We concluded that the mutation-harboring cancer cells could be stained and discriminated on a single-cell microarray chip because the NCI-H1975 cells had a fluorescence intensity twice that of the negative control (A549) cells. As the maximum fluorescence intensity of A549 was approximately 80–100, the cells with a fluorescence intensity of greater than 100 in this study were classified as positive (NCI-H1975) cells, and the cells with a fluorescence intensity of 100 or lower were classified as A549 cells. The differences of fluorescent intensity of NCI-H1975 and A549 cell lines were enough to discriminate each other in single-cell microarray chip under the fluorescence microscope combined with PNA-DNA probe technology. Therefore, these results strongly indicated the imaging analysis of single nucleotide-mutated single-cancer cells can be achieved. To our knowledge, there have been no reports confirming that lung cancer cells containing the mutated and non-mutated cells have been aligned and analyzed in single cells using a microarray. The single-cell microarray chip technology in this research has made it possible to easily and accurately separate and analyze the mutation-harboring cancer cells at the single cell level. Various cancer cells can be separated into single cells, regardless of their type, suggesting that single-cell microarray chip technology could be used for the analysis and diagnosis of a wide range of cancers.

### 3.2. Detection of T790M-Mutated Cancer Cells Using the Combined Technologies of the Single-Cell Microarray Chip and the PNA-DNA Probe

**Figure 3:**
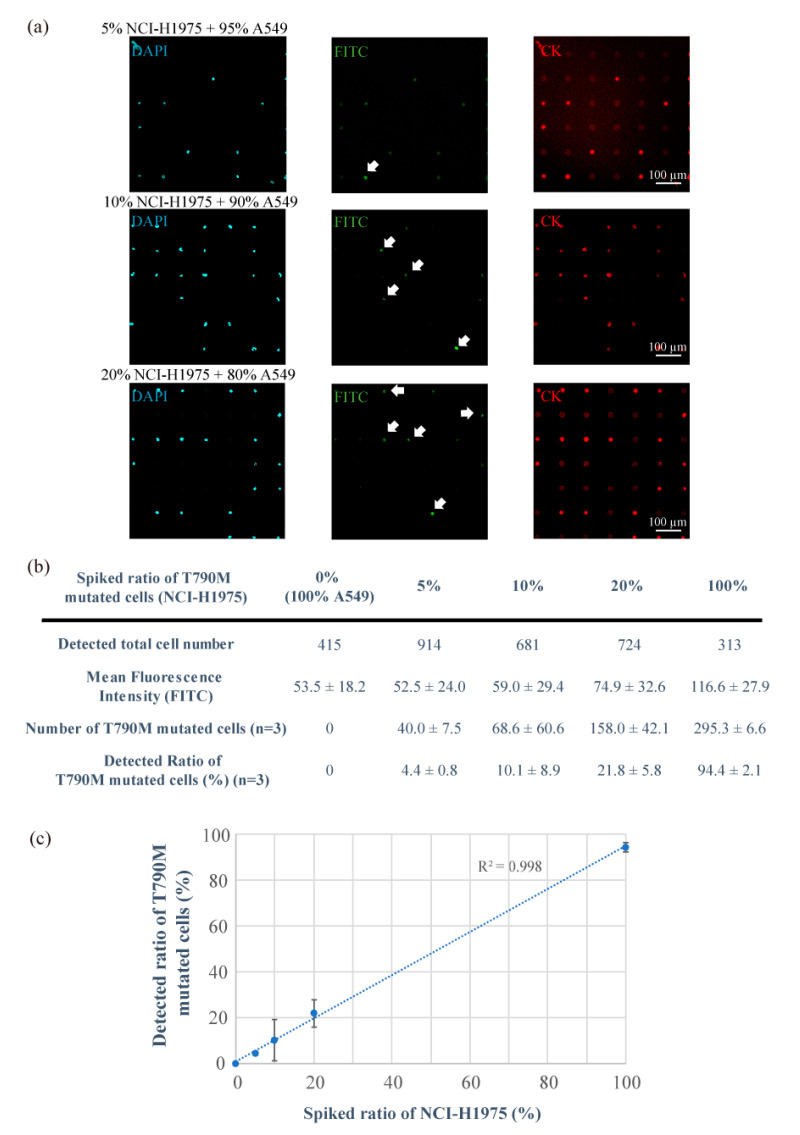
Analysis of the ratio of T790M-mutated cancer cells (NCI-H1975) spiked into non-mutated cells (A549) using combination technology with the single-cell microarray chip and peptide nucleic acid (PNA)-DNA probe. (**a**) Representative fluorescent images of 5%, 10%, and 20% NCI-H1975 cells spiking into A549 cells stained with diluted 4,6-diamidino-2-phenylindole (DAPI; blue), fluorescein isothiocyanate (FITC)-conjugated PNA-DNA probes (green), and phycoerythrin (PE)-labeled cytokeratin (CK) antibody (red). White arrows indicate positive cells (T790M-mutated). (**b**) Information on the number and ratio of T790M-mutated cells (NCI-H1975) spiked into A549 cells. (**c**) Linearity plots for the spiked and detected ratio of T790M-mutated cells (NCI-H1975) spiked into A549 cells. R^2^ indicates the correlation coefficient. The results are shown as the mean ± standard error of the three replicates.

For gene mutation analysis using the most versatile system, the NGS system, the system generally requires more than 20% of cells within the sample to have the target mutation [44,45]. In this study, to examine the sensitivity of our detection system, 5%, 10%, or 20% NCI-H1975 (T790M-mutated cancer cell line) cells were spiked into A549 cells and dispersed onto a single-cell microarray chip. The NCI-H1975 cell were identified after the cells were dispersed in single-cell microarray chip. The cells were stained by DAPI, CK, and PNA-DNA probes on the chip. All single-cancer cells (mutated and non-mutated) were filled into 50–60% of the microchambers on the single-cell microarray chip and stained with DAPI and CK, without dislodging the cells from the microchambers by the washing process (Figure 3a). In addition, spiked ratio of cell samples did not appear to change before and after dispersing on the single-cell microarray chip because two types of lung cancer cells were separated into single cells on the chip in the same way after the washing process (Figure 2). This suggested that multiple stainings at the single-cell level on the single-cell microarray chip had been achieved.

The target T790M-mutated single-cancer cells could be stained by FITC-conjugated PNA-DNA probes depending on the spiked ratio of mutated cancer cells (Figure 3a). We calculated the fluorescence intensity of the T790M-mutated cells that were observed on the single-cell microarray chip and counted the number of cells having a fluorescence intensity of not less than 100, which is the maximum intensity of the negative control cells (A549). There were 40 ± 7.5 (4.4 ± 0.8%), 68 ± 60 (10.1 ± 8.9%), and 158 ± 42 (21.8 ± 5.8%) positive cells in the 5%, 10%, and 20% NCI-H1975 samples, respectively (Figure 3b,c). The theoretical cell numbers of NCI-H1975 were 45.7, 68.1, and 144.8 cells in the 5%, 10%, and 20% NCI-H1975 samples, respectively. Therefore, the 5%, 10% and 20% mutant cell samples were exactly proportional to the theoretical ratio. The results show that this system can detect the target mutated single-cancer cells containing 5% or higher ratio. There were no positive cells in the 0% NCI-H1975 (100% A549) samples. Therefore, the positive cells detected in this study had a high probability of being NCI-H1975 cells. Figure 3c also indicates that each spiked ratio of T790M-mutated cells showed a strong linearity, and the coefficient of determination was 0.998. Some data appear to vary because of the sensitivity, specificity, and cell transduction efficiency of the PNA-DNA probe. It can sometimes cause difficulties in accurate measurement, especially in a low ratio of target single cells. However, improved PNA-DNA probes will enable more sensitive and selective measurement of single nucleotide-mutated cells on single-cell microarray chips. Furthermore, for higher single-cell occupancy and sensitive detection, it is also possible to accurately detect lower ratio of mutated cells by improving the microchamber design of the single-cell microarray chip.

The NGS system, which is currently used in the accurate whole-genome sequencing of cancer cells and identifying anticancer drug-resistant cells, requires expensive equipment and a high level of expertise. In addition, accurate analysis is difficult unless over 20% of cancer cells in the sample contain the target mutation [43,44,45]. Therefore, it is difficult to preemptively diagnose EGFR-mutated cells with NGS using only a few gene-mutated cells collected from the small amount of lung-cancerous tissues. The NGS system generally needs the DNA sample at least 50–1000 ng [43], which means almost 1.5 × 10^4^–1.5 × 10^6^ cells are required. On the other hand, the single-cell microarray chips system required total 1 × 10^6^–5 × 10^6^ cells. The number of cells (sample volume) required for analysis is almost the same level. However, we can further reduce the number of cells by improving the design of microarray chip with higher integrated microchambers. The real-time PCR-based analyzing systems also have high sensitivity, but these conventional methods also require expensive equipment, time-consuming detection (3–5 h for typical PCR systems), and expert technical knowhow. In contrast, the single-cell microarray chip and PNA-DNA probe technology method was able to detect 5–20% of the mutated cells, and the process was completed within 1 h, including cell preparation. This method has been found to be faster and more sensitive than the NGS system because it is easy to use and can be measured using basic fluorescence microscopes. Although the sensitivity of the combined single-cell microarray chip and PNA-DNA probe system is similar to that of several real-time PCR-based methods, it appears that the former system is superior to the latter in terms of detection time and user-friendliness.

Therefore, this combined technology of single-cell microarray chips and PNA-DNA probes system could be a useful tool for analyzing cancer tissue containing a small number of single nucleotide-mutated single-cancer cells collected by biopsy before and after the anticancer drug treatment. In addition, by changing the sequence of PNA-DNA probes to various target mutations, it is possible to detect multiple gene-mutated cancer cells at the single-cell level and effectively select an anticancer agent. A further advantage of the single-cell microarray chip system is the ability to recover target single cells. Previously, we reported the recovery of single-cells by using a micromanipulator and a single-cell microarray chip [7]. Although we did not recover the T790M-mutated single-cancer cells from the microchambers in the present work, for further analysis it would also be easy to recover the mutated single-cancer cells by using a micromanipulator system in the future.

Recently, minimally invasive diagnostic methods before surgery are strongly required. The liquid biopsies, targeting circulating tumor cell (CTC) or circulating tumor DNA (ctDNA) in the bloodstream are promising diagnostic methods for the prognosis of metastatic cancer. The combined technology of single-cell microarray chips and PNA-DNA probes could also be applicable for the liquid biopsies that require the analysis of a small number of CTCs or ctDNAs from whole blood samples. Although the single-cell microarray chip can detect only 5% target single-cells at this stage, by improving the design of microarray chip with higher integrated microchambers, it will be easy to detect the rare cancer cells like CTCs from larger numbers of cells. Therefore, it is strongly suggested that this method of analysis be used as a new diagnostic tool for cancer by detecting cancer tissue or liquid biopsy samples at single-cell level.

## 4. Conclusions

In this study, we examined the possibility of analyzing the single nucleotide-mutated cancer cells at the single cell level using the combined technologies of single-cell microarray chips and PNA-DNA probes. Screening for rare lung cancer cells harboring the T790M mutation in the EGFR sequence showed that the T790M-mutated cancer cells had a strong fluorescent signal, which was observed in proportion to the spiking concentrations in the non-mutated cancer cells. The combined technology of single-cell microarray chips and PNA-DNA probes makes it possible to analyze a small number of single nucleotide-mutated single-cancer cells from a large number of non-mutated cancer cells. These results indicate that this technology can effectively screen for drug-resistant cancer cells. In the future, by further improving the sensitivity and specificity of the PNA-DNA probes, we will be able to detect, with high sensitivity, various gene-mutated anticancer drug-resistant cells from cancer tissue or liquid biopsies samples collected from cancer patients.
